# Hospital-Acquired Infections Caused by Carbapenem-Resistant Enterobacteriaceae: An Observational Study

**DOI:** 10.3390/microorganisms11061595

**Published:** 2023-06-16

**Authors:** Hamzah J. Aldali, Azra Khan, Abdullah A. Alshehri, Jehad A. Aldali, Sultan Ayoub Meo, Ali Hindi, Emadeldin M. Elsokkary

**Affiliations:** 1 Cellular and Molecular Medicine, College of Biomedical Science, University of Bristol, Bristol BS8 1DT, UK; 2 School of Life Sciences, Faculty of Health & Life Sciences, Coventry University, Coventry CV2 2DX, UK; 3 Department of Clinical Pharmacy, College of Pharmacy, Taif University, Al Huwaya, Taif 26571, Saudi Arabia; 4 Department of Pathology, College of Medicine, Imam Mohammad Ibn Saud Islamic University, Riyadh 13317, Saudi Arabia; 5 Department of Physiology, College of Medicine, King Saud University, Riyadh 11461, Saudi Arabia; 6 Division of Pharmacy and Optometry, School of Health Sciences, Faculty of Biology, Medicine and Health, The University of Manchester, Manchester M13 9PT, UK; 7 Department of Psychology, Imam Mohammad Ibn Saud Islamic University, Riyadh 13317, Saudi Arabia

**Keywords:** hospital-acquired infection, Enterobacteriaceae, carbapenem-resistant

## Abstract

Worldwide, hospital-acquired infections (HAIs) are continuously rising within healthcare settings, leading to high mortality and morbidity rates. Many hospitals have reported the spread of carbapenemases globally, specifically within the *E. coli* and *K. pneumoniae* species. This study was aimed at analyzing the state of hospital-acquired, carbapenem-resistant *E. coli* and *K. pneumoniae* in the United Kingdom between 2009 and 2021. Moreover, the study analyzed the most efficacious approaches to patient management for controlling the carbapenem-resistant Enterobacteriaceae (CRE) spread. Initially, 1094 articles were identified as relevant for screening, and among them, 49 papers were eligible for full-text screening, with a total of 14 articles meeting the inclusion criteria. The information was recorded from published articles through PubMed, the Web of Science, Scopus, Science Direct, and the Cochrane library and was used to search for hospital-acquired carbapenem-resistant *E. coli* and *K pneumoniae* in the UK between 2009 and 2021, in order to evaluate the spread of CRE in hospitals. The total number of carbapenem-resistant *E. coli* was 1083 and this was 2053 for carbapenem-resistant *K. pneumoniae* in more than 63 UK hospitals. KPC was the dominant carbapenemase produced by *K. pneumoniae*. The results showed that the treatment options considered depended on the type of carbapenemase produced; *K. pneumoniae* showed more resistance to a treatment options, i.e., Colistin, than the other carbapenemase. The current state of the UK is at minimal risk for a CRE outbreak; however, appropriate treatment and infection control measures are highly required to prevent this CRE spread at the regional and global levels. The present study findings have an important message for physicians, healthcare workers, and policymakers about hospital-acquired carbapenem-resistant *E. coli* and *K. pneumoniae* spread and approaches to patient management.

## 1. Introduction

A hospital-acquired infection (HAI) is an infection that is acquired in a healthcare setting within two days of admission [1]. The threat of HAIs is continuously increasing, causing an estimated 2.5 million new cases every year in Europe, 500,000 of which are caused by multi-drug resistant (MDR) microorganisms [2]. Gram-negative bacilli studies have revealed that seasonal hospitalized patients with bloodstream infections are caused by many bacteria, including *E. coli* and *Klebsiella* spp. (both are species within the Enterobacteriaceae family), and these infections are at their peak during the summer in North America, the Middle East, Europe, Asia, and Australia [3]. The majority of these bacterial infections are MDR species, which are resistant to many known antibiotics, leading to more than 25,000 deaths per year in European countries [4].

Recently, Enterobacteriaceae species were shown to be highly resistant to broad-spectrum antibiotics through extended-spectrum β-lactamases (ESBLs), due to the acquisition of gene-encoding enzymes, which upregulate efflux pumps and alterations to the antibiotic binding sites [5]. Therefore, carbapenems were considered to be the antibiotic of choice to treat MDR Enterobacteriaceae [6]. However, many bacterial strains from the Enterobacteriaceae family are becoming carbapenem-resistant Enterobacteriaceae (CRE). Three major carbapenemases are considered to be epidemiologically and clinically significant: *Klebsiella pneumonia* carbapenemase (KPC), New Delhi metallo-β-lactamase (NDM), and oxacillinase (OXA), which hydrolyze carbapenem [7,8]. These carbapenemases are commonly found in the *K. pneumoniae* and *E. coli* species [9].

*K. pneumoniae* has been reported as the leading cause of bacteremia and pneumonia in the United Kingdom (UK) [10]. Currently, carbapenem-resistant *K. pneumoniae* (CRKP) is a major health-related pathogen responsible for pneumonia, surgical site infection, bloodstream infection, UTIs, and meningitis, leading to high morbidity and mortality rates [11,12]. *E. coli* and *Klebsiella* are the leading causes of hospital-acquired infections worldwide [13]. In previous decades, *E. coli* became increasingly resistant to β-lactams and the carriage of resistant AmpC plasmids [14]. Carbapenem was again considered to be the gold standard for treating nosocomial *E. coli* infections. However, carbapenem-resistant *E. coli* started to emerge, mainly through the production of carbapenemases, along with a decreased antibiotic permeability (increased efflux pump and lack of porin presence) and an alternation of the carbapenem binding sites [15,16].

Despite the wide spread of CRE strains, specifically carbapenem-resistant *K. pneumoniae* (CRKP) and CRE *E. coli*, a detailed examination of the spread of CRE strains in the UK in the past decade appears to be limited in the literature. Therefore, this study investigates the spread of CRKP and CRE *E. coli* in the UK from 2009 to 2021. Additionally, it identifies the most common type of carbapenemases carried by CRKP and CRE *E. coli* and the antibiotic treatments used, depending on the type of carried carbapenemase.

## 2. Material and Methods

### 2.1. Study Design and Settings

The present data-based observational study was conducted under the College of Biomedical Science, University of Bristol, Bristol, United Kingdom and School of Life Sciences, Faculty of Health & Life Sciences, Coventry University, Coventry University, United Kingdom and College of Medicine, King Saud University, Riyadh, Saudi Arabia during the period from January to October 2022.

### 2.2. Protocol and Registration

For the search of the literature, the Preferred Reporting Items for Systematic and Meta-Analysis (PRISMA) guidelines were followed while conducting this study, in order to select the reliable sources that correlated with the research aims. The protocol was registered on PROSPERO (registration number CRD42022303741).

### 2.3. Search Strategy

In this study, data were retrieved about the incidence and prevalence of hospital-acquired carbapenem-resistant *E. coli* and *K. pneumoniae* to investigate the most effective methods for managing HA-CRE. Cases of CRE in the UK published in scientific journals between 2009 and 2021 were used to evaluate the spread of CRE in hospitals. Many studies started HA-CRE data collection in UK hospitals in 2009; thus, 2009 was set as the starting line of this study. The minimum inhibitory concentration (MIC) antibiotic treatment options were analyzed to identify the resistant and susceptible strains, depending on the type of carbapenemase, NDM, KPC, OXA, VIM, and IMP produced by the reported CRE *E. coli* and *K. pneumoniae*.

### 2.4. Eligibility Criteria

The primary sources of the reported carbapenem cases were collected using scientific search engines. The inclusion and exclusion criteria determined the papers that were included or rejected for the review and data collection. The included studies were those published in the UK that contained reported cases, quantitative records of CRE-infected patients, and *E. coli* and *K. pneumoniae* within the CRE species. In contrast, studies performed on non-UK reported cases, data reported before 2009, carbapenem-susceptible Enterobacteriaceae, data that did not specify the Enterobacteriaceae species, and community-acquired infections were all excluded. Moreover, brief communications, editorials, and review articles were also excluded.

### 2.5. Study Selection

This study analyzed the published literature and research articles on reported patients who acquired carbapenem-resistant *E. coli* and/or *K. pneumoniae* during >2 days of a hospital stay in the UK between 2009 and 2021. The literature was searched using scientific research engines, including PubMed, the Web of Science, Science Direct, Scopus, and the Cochrane Library to find the relevant articles. The search terms used were the Boolean operators ‘AND’, ‘OR’, and ‘NOT’ and included ‘carbapenem-resistant Enterobacteriaceae’ OR ‘Carbapenem-resistant *Escherichia coli’* OR ‘Carbapenem-resistant *Klebsiella pneumoniae*’ AND ‘Hospital-acquired infection’ OR ‘nosocomial infection’ OR ‘healthcare-acquired infection’ AND ‘United Kingdom’ OR ‘England’ OR ‘Wales’ OR ‘Northern Ireland’ OR ‘Scotland’. Furthermore, the scope of the search was expanded by screening the reference list of the relevant articles to identify topic-related articles. Only full-text articles published in the English language were included, and, following the removal of duplicated articles, the selection of the papers was based on three sections: (1) titles, (2) abstracts, and (3) results and full-text screening.

### 2.6. Data Recording

At the post-inclusion and exclusion stage, Excel and EndNote were used to uniquely select the relevant data and identify which factors were presented by each article. Each article was presented in a row on an Excel sheet to represent the information provided by the article. The columns presented the total number of CRE reported cases, the hospital location, the specific quantity of carbapenem-resistant *E. coli* and *K. pneumoniae*, the type of carbapenemase, the sample type (blood, urine, or wound swap, etc.), and the age group. However, the articles that presented MIC values from patient-identified CRE isolates were separately recorded using the Excel sheet, followed by a total summary of all the values from the correlated papers for all the antibiotics. For each antibiotic, the susceptibility, intermediate, and resistance breakpoint values were determined using the European Society of Clinical Microbiology and Infectious Diseases (EUCAST) breakpoint tables.

### 2.7. Data Analysis and Ethical Approval

The statistical analyses were performed separately for both *E. coli* and *K. pneumoniae* using Statistics Package Social Science (SPSS) Version 25 (IBM Armonk, New York, NY, USA). Statistical results with a *p*-value below 0.05 were considered as significant. Additionally, the percentage, frequency, mean, and median were calculated. The data conducted are variables of numerical data, which are non-continuous discrete values. A chi-squared (χ^2^) analysis was performed, using SPSS Version 25, with the following formulae: $Er,c=\frac{Nr-Nc}{n}$ and $\chi^{2}=\Sigma \frac{{\left(Or,c-Er,c\right)}^{2}}{n}$. The Chi-square test involved the presentation of the observed count and the expected count, followed by the corresponding chi-square value. The residual values were utilized to indicate the disparity between the observed and expected value. Moreover, the chi-square value and degree of freedom provided the *p*-value, which was necessary for determining whether to reject the null hypothesis or not. In this study, data were retrieved from the publicly available literature, hence ethical approval was not required.

## 3. Results

**Figure 1:**
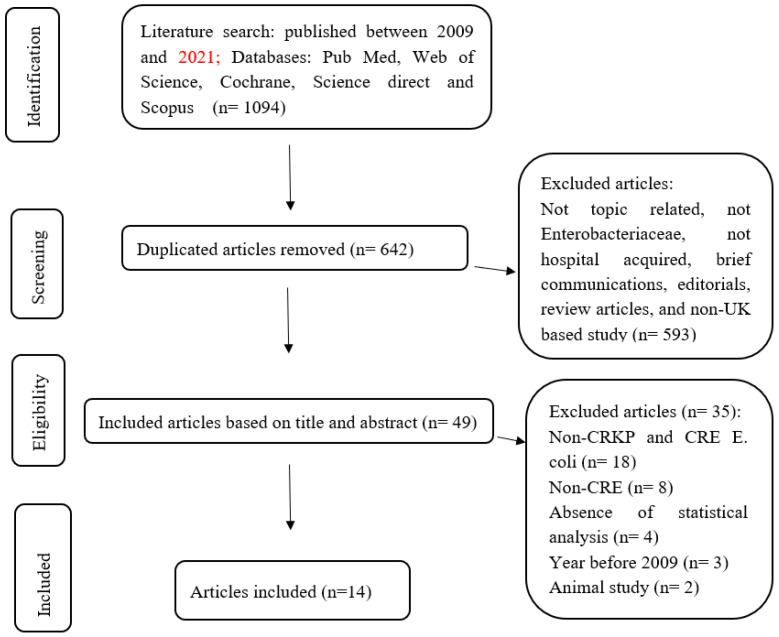
Flow diagram of the search process and the number of selected and rejected studies.

During the literature search, 1094 retrieved articles were identified as relevant for screening, and among them, 49 papers were eligible for full-text screening, with a total of 14 articles [17,18,19,20,21,22,23,24,25,26,27,28,29,30] meeting the inclusion criteria (Figure 1).

**Figure 2:**
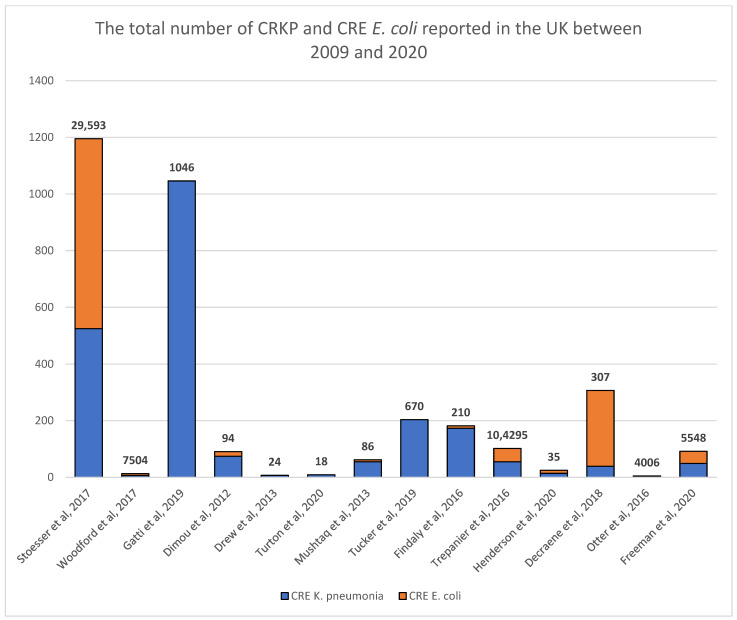
Specimen samples are presented above each study that was collected to identify the number of CRE *K. pneumoniae* (in blue) and CRE *E. coli* (in orange) [17,18,19,20,21,22,23,24,25,26,27,28,29,30].

These articles extracted different specimens from thousands of hospitalized patients and recorded the numbers of hospital-acquired CRKP and CRE *E. coli* infections (Figure 2). From the included articles, 1083 contained CRE *E. coli* and 2053 contained CRKP from more than 63 patients hospitalized for more than two days in a UK hospital between 2009 and 2021. Three studies [17,18,19] reported CRKP but not CRE *E. coli*. In contrast, for those who identified both resistant species, only four studies from three hospitals in Manchester showed a higher percentage of patients with CRE *E. coli* than CRKP.

The chi-square result for the observed data was 239.24 with one degree of freedom, and the *p*-value was <0.001 (Table 1). These results indicate a significant difference between the observed and expected frequencies of *E. coli* and CRKP.

### 3.1. Spread of CRE from Reported Hospitals

**Figure 3:**
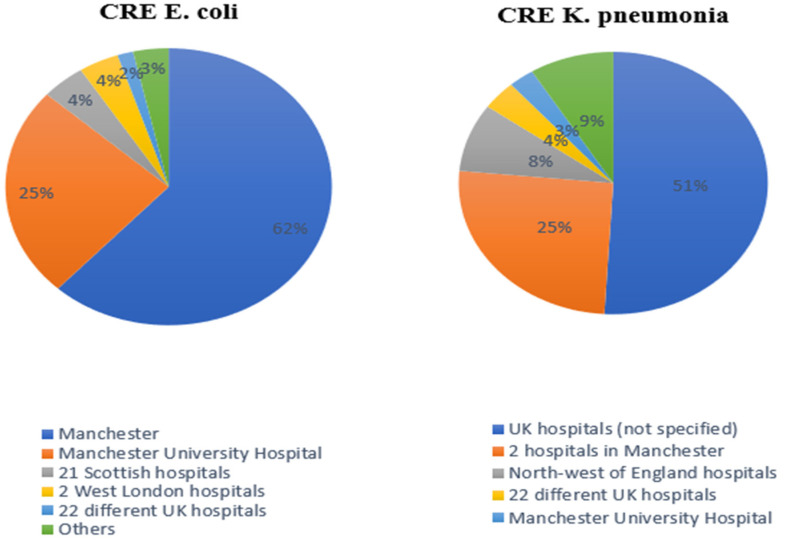
The reported percentage of both CRE *E. coli* and CRKP depends on the location of UK hospitals. The 22 reported hospitals were collected from one study [26]. Others represent the remainder percentage from different cities around the UK.

Different hospitals reported CRE *E. coli* and CRKP, some of which had one specific location, while others were from multiple unspecified hospitals around the UK (Figure 3).

The majority of CRE *E. coli* cases identified in Manchester hospitals were reported over five and eight years of monitoring HA-CRE [23,25]. In contrast, 51% percent of CRKP cases were reported by a 9-year retrospective study across multiple hospitals in the UK.

### 3.2. Major Carbapenemase Plasmids

Eleven studies [17,18,20,21,22,23,24,25,26,28,29] quantitatively reported the type of carbapenemase, mainly KPC, NDM, and OXA-48-like, produced by either CRE *E. coli* or CRKP. The total number of reported CRKP and CRE *E. coli* cases carrying one of the major carbapenemases was 2756, 35% of which were CRE *E. coli* and 65% were CRKP (Table 2).

The chi-square result for the observed data was 2301.87 with two degrees of freedom, and the *p*-value was <0.001 (Table 3). These results indicate a significant difference between the observed and expected frequencies of KPC plasmid, OXA-48-Like plasmid, and NDM.

The chi-square test for the observed data yielded a result of 1403.91 with two degrees of freedom, and the *p*-value was <0.001, indicating a significant difference between the plasmid types carried by the *E. coli* and CRKP (Table 4). The percentage of KPC plasmid in CRKP (84.6%) was much higher than that in *E. coli* (15.4%), while OXA-48-Like plasmid in *E. coli* (94.3%) was much higher, compared to only 5.7% in CRKP.

### 3.3. Minimum Inhibitory Concentration Calculations

**Figure 4:**
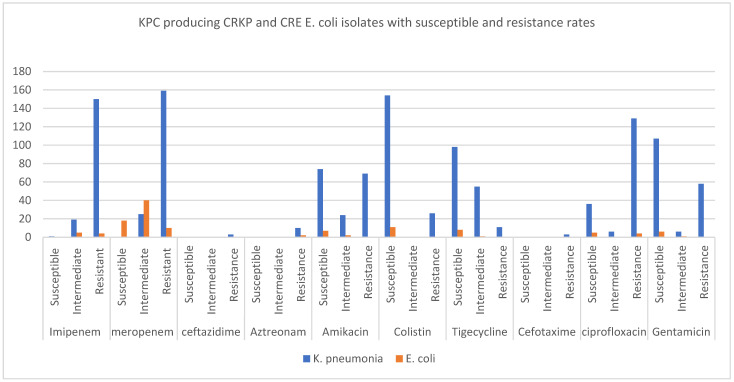
The MIC of ten different antibiotics were identified for multiple isolates of KPC-producing *K. pneumoniae* and *E. coli* to identify the number of susceptible, intermediate, and resistant isolates.

**Figure 5:**
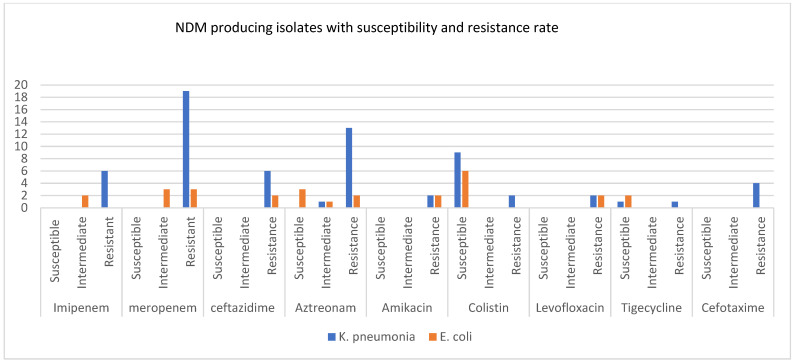
The graph shows susceptibility, intermediate, and resistant isolates of NDM-producing *K. pneumoniae* and *E. coli* for nine different antibiotics.

**Figure 6:**
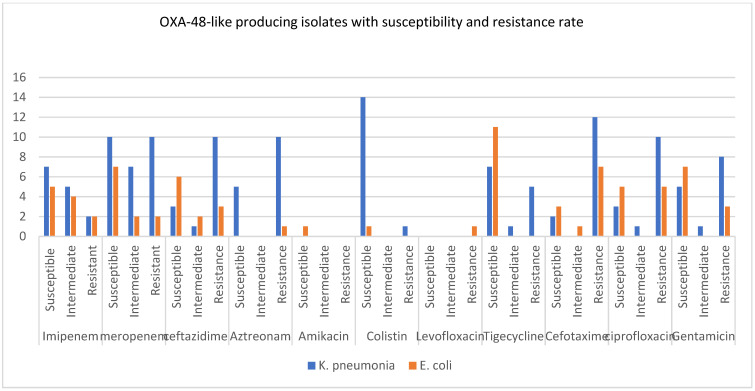
This chart shows the susceptibility, intermediate, and resistance rates of OXA-48-like produced carbapenemase by *K. pneumoniae* and *E. coli* against 11 antibiotics.

The MICs of different classes of antibiotics, including carbapenem, cephalosporins, aminoglycosides, quinolones, and tetracycline, were shown in six studies in order to promote the resistance and susceptibility of these antibiotics against carbapenemase-producing CRKP and CRE *E. coli*, specifically KPC, NDM, and OXA-48 carbapenemases (Figure 4, Figure 5 and Figure 6).

As shown in Figure 4, approximately 150 different isolates of KPC-producing hospital-acquired CRKP were shown to be largely resistant to carbapenem and ciprofloxacin antibiotics, but susceptible to colistin (antimicrobial peptide) and tigecycline. In contrast, the MICs of KPC-carrying *E. coli* against antibiotics were not highly reported in comparison to KPC-producing CRKP. However, all the KPC-producing *E. coli* isolates were susceptible to colistin, similar to tigecycline, gentamicin, and amikacin.

The percentage of NDM-carrying CRE was less than 1% for both CRKP and CRE *E. coli*. However, Drew et al., Mushtaq et al., and Woodfield et al. [17,20,28] all presented the MICs against carbapenems, cephalosporins, aminoglycoside, tetracycline, and quinolone antibiotics (Figure 5). NDM-producing CRKP showed a high resistance against meropenem, ceftazidime, cefotaxime, aztreonam, ciprofloxacin, and gentamicin. In contrast, a susceptibility to colistin and tigecycline was observed. Additionally, NDM-carrying *E. coli* was fully susceptible to colistin and tigecycline.

Finally, the second most commonly spread carbapenemase between CRE *E. coli* and CRKP is the OXA-48-like carbapenemase, which showed high susceptibility rates, with few isolates showing resistance against carbapenems, colistin, and tigecycline (Figure 6). However, more OXA-48-producing CRKP isolates were resistant to meropenem, ceftazidime, aztreonam, cefotaxime, ciprofloxacin, and gentamicin.

## 4. Discussion

This study aimed at presenting the state of CRKP and CRE *E. coli* in the UK between 2009 and 2021. This was followed by an investigation into the spread of the three most common carbapenemases (NDM, KPC, and OXA carbapenemases) and the type of antibiotic treatments that were used to treat the infected patients. This study specifically focused on the two Enterobacteriaceae species that are most likely to spread within hospitals worldwide, CRKP and CRE. *E. coli*, with a detailed analysis of fourteen different articles. Different specimens were taken, including blood, urine, and sputum, from hospitalized patients, which showed that CRKP was approximately twice as prevalent as CRE *E. coli*. According to the European Centre for Disease Prevention and Control, both CRKP and CRE *E. coli* are detected by less than 1% in the UK and their spread is regional [31]. However, there has been an increase in the number of CRE-reported cases each year, from three cases in 2003 to 1893 cases in 2015 [32]. Additionally, similar to this study, a study was performed to identify the rate of CRE species in London, which showed that CRKP was the most common species [33]. Although there has been an increase in the rate of carbapenem-producing *E. coli* in the UK, there are still fewer than one hundred cases per year [34]. The spread of KPC-carrying CRKP is commonly reported as a multidrug-resistant nosocomial species. Our results showed that KPC-carrying *K. pneumoniae* is less susceptible to antimicrobials compared to the other plasmids carried by *K. pneumoniae*. It is thought that the infection control measures put in place, such as strict patient cohorts, the correct choice of treatment, and only using the antimicrobial peptide colistin as a last resort treatment option, are the reasons that the spread of such a resistant strain has been controlled thus far [35].

It was observed that high percentages of both CRKP and CRE *E. coli* cases were found in Manchester. The city of Manchester showed an estimated 10× higher detection rate of CRE compared to other areas of England [36]. Similar to the results of the presented study, an epidemiological study performed to report the prevalence of carbapenem-resistant plasmids in England showed that Manchester was a hotspot for KPC- and OXA-48-like-producing CRE, which reported a large number of carabapenem resistant Enterobacteriaceae cases in the northwest of England [36].

Depending on the type of carbapenemase produced by Enterobacteriaceae, the treatment and management options differ. It was observed from the studies that the NDM, IMP, and VIM carbapenemases are minority groups, being less than 1% of the total reported HA-CRE. Public Health of England reported more than 100 NDM-producing CRE isolates between 2012 and 2015 [32]. The reason behind the small number of NDM-carrier reports may be that the majority of the selected studies for this study were published before 2015. Therefore, further investigation of the current NDM spread is required, as the UK was one of the earliest countries to report NDM carbapenemase [37]. Additionally, it has been previously reported that *K. pneumoniae* is the most commonly reported isolate that produces carbapenemases through plasmid uptake, specifically KPC-carrying plasmids [38]. Only one protracted outbreak caused by OXA-48-like-producing *K. pneumoniae* was recorded in the UK between 2008 and 2010 [39]. The outbreak, which was in a west London Hospital, was brought under control by strict IPC procedures, including improving screening, raising nursing staff awareness, improving laboratory detection mainly via PCR, and the judicial use of antimicrobials following antimicrobial guidelines [39].

Six out of the fourteen collated studies investigated the MICs of antibiotics in CRE-infected patients. The treatment options using antibiotics were examined for more than 500 patients. Although antibiotic treatment options are very limited against MDR bacteria, it was observed that some CRE were susceptible to more than one antibiotic. Antibiotics such as tigecycline, aminoglycosides, and the antimicrobial peptide colistin were described as the optimal treatments for MDR Gram-negative infections [40]. Due to the limited number of patients with IMP, VIM, and NDM found by this study, it was difficult to determine a suitable treatment method against these particular carbapenemases. CRKP appears to be highly resistant to carbapenems compared to KPC-producing *E. coli*. However, both species showed a high percentage of susceptibility to tigecycline, colistin, and gentamicin. OXA-48-like remains susceptible to many antibiotics, including broad-spectrum cephalosporins [41]. Moreover, OXA-48s are unique carbapenemases with a low hydrolytic function toward meropenem and imipenem [42]. As observed, the susceptibilities of colistin and tigecycline are high; therefore, combination therapy is suggested to reduce both mortality and morbidity rates. This susceptibility profile shows a positive sign for managing plasmid-carrying Carbapenem-resistant *E. coli* and *K. pneumoniae*.

Although there has been an upsurge in the rate of carbapenem-producing *E. coli* in the UK, there is still a limited number of cases reported, which is less than 100 cases per annum [34]. It is believed that stringent infection control measures must be put in place, such as strict patient cohorts, the correct choice of treatment modalities, and using colistin as a last resort treatment option, which are the reasons that the spread of such a highly resistant strain has been controlled [35]. However, contracting a resistant strain, such as CRE, can lead to prolonged patient stays in hospitals. Therefore, maintaining good infection prevention practices is vital for reducing transmission rates. Healthcare staff members are required to ensure that a sterile environment is created by preventing contamination from body fluids, using sterile equipment and safe injection practices, and ensuring respiratory hygiene in order to prevent ventilator-associated pneumonia [43]. Moreover, it is advised that patients with known respiratory tract infections are placed within a controlled ventilation room with air filters to prevent suspended droplets from being passed around the hospital by the medical staff environment.

To limit the spread of threatening CRE infections, 39 European countries, including the UK, agreed to minimize the burden of CRE infection by keeping recorded CRE-positive patient data between the European Centre for Disease Prevention (ECDP) and Control in Stockholm [44]. Previously, in 1991, the UK government decided to direct the National Health Service (NHS) to further develop hospital care facility management, as the design of the hospital can significantly affect its HAI rates [45]. Public Health England states that hospitals must screen high-risk patients that have previously been infected by CRE and they should be placed in isolation after readmission [32]. These patients should then be entered into Public Health England’s microbiological database, including details such as their demographic and specimen data and treatment progression approaches.

## 5. Strengths and Limitations

To the authors’ knowledge, this is the first comprehensive study which analyzed the published data for both CRE *E. coli* and CRKP, with the identification of carbapenemase, KPC, NDM, and OXA-48. This study included data from more than 60 hospitals around the UK since 2009, which made this study more specific for the identification of areas with high levels of infection. Furthermore, a selective identification of the most common carbapenemase in each species provided an overview of the most common and most resistant types of carbapenemase.

Its limitations, by contrast, include the fact that a few articles were CRKP-specific and did not investigate the presence of HA-CRE *E. coli*. Additionally, very limited data were available regarding the IMP, VIM, and NDM carbapenemases. Furthermore, the majority of the articles were published based on the data from hospitals in England, and the extent of the CRE spread in hospitals in Scotland, Wales, and Northern Ireland is currently unclear. Therefore, a future investigation into the CRE spread in these geographical areas is required.

## 6. Conclusions

This study provided the current CRE *E. coli* and CRKP situation from more than 60 hospitals and identified 11 different antibiotics that could be used for the treatment of CRE, thus providing a summary of the potential treatment options, depending on the type of carbapenemases carried. The study’s findings reveal a low risk of CRE outbreak; however, appropriate treatment, alongside appropriate infection control measures, is highly required to prevent this CRE spread. The study’s findings can be used to inform the IPC measures taken by the UK government to prevent future outbreaks. However, it is worth noting that further studies may be required to confirm these findings and ensure the effectiveness of the identified treatment options. Moreover, such studies should be conducted worldwide, including in the USA, Europe, Asia, and the Middle East, to understand and prevent the spread of CRE infection.

## Figures and Tables

**Table 1:** Observed and expected frequencies of *E. coli* and CRKP with Chi-Square results.

	Observed N	Expected N	Residual	Chi-Square	df	*p*-Value
*E. coli*	972	1378.0	-406.0	239.24	1	<0.001
Carbapenem resistant *K. pneumoniae* (CRKP)	1784	1378.0	406.0			
Total	2756					

**Table 2:** The collected isolates with c from the 14 studies.

	KPC	OXA-48-Like	NDM	Total
*E. coli*	314	648	10	972
CRKP	1729	39	16	1784
Total column	2043	687	26	2756

**Table 3:** Chi-square results of observed KPC, OXA-48-Like, and NDM plasmids.

	Observed N	Expected N	Residual	Chi-Square	df	*p*-Value
KPC	2043	918.7	1124.3	2301.87	2	<0.0001
OXA-48-Like	687	918.7	-231.7			
NDM	26	918.7	-892.7			
Total	2756					

**Table 4:** Plasmid Types Carried by *E. coli* and Carbapenem-Resistant *K. pneumoniae* (CRKP) with Chi-Square Results.

			Type			Total	Pearson Chi-Square	df	*p*-Value
			KPC	OXA-48-Like	NDM				
Category	***E. coli***	**Count**	**314**	**648**	**10**	972	1403.91	2	<0.001
		% within Type	15.4%	94.3%	38.5%	35.3%			
	CRKP	Count	1729	39	16	1784			
		% within Type	84.6%	5.7%	61.5%	64.7%			
Total		Count	2043	687	26	2756			
		% within Type	100.0%	100.0%	100.0%	100.0%			

## Data Availability

Not applicable.
